# Point prevalence surveys of antibiotic prescribing in children at a tertiary hospital in a resource constraint, low-income sub-Saharan African country—the impact of an antimicrobial stewardship program

**DOI:** 10.1186/s12887-024-04847-3

**Published:** 2024-06-04

**Authors:** Patricia Akintan, Philip Oshun, Chioma Osuagwu, Olafoyekemi Ola-bello, Iretiola Fajolu, Alero Roberts, Edamisan Temiye, Oyinlola Oduyebo

**Affiliations:** 1 Department of Paediatric College of Medicine, University of Lagos, Lagos, Nigeria; 2 Department of Paediatrics, Lagos University Teaching Hospital, Idi-Araba, Lagos, Nigeria; 3 Department of Medical Microbiology and Parasitology College of Medicine, University of Lagos, Lagos, Nigeria; 4 Department of Medical Microbiology and Parasitology, Lagos University Teaching Hospital, Idi-Araba, Lagos, Nigeria; 5 Department of Community Health and Primary Care, College of Medicine, University of Lagos, Lagos, Nigeria; 6 Department of Community Health and Primary Care, Lagos University Teaching Hospital, Idi-Araba, Lagos, Nigeria

**Keywords:** Antimicrobial, Prescribing, Stewardship, PPS, Children

## Abstract

**Background:**

Resistance to multiple antibiotics by several pathogens has been widely described in children and has become a global health emergency. This is due to increased use by parents, caregivers, and healthcare providers. This study aims to describe the prevalence rates of antibiotic prescribing, ascertain the impact of antimicrobial stewardship programs, and target improving the quality of antibiotic prescribing in the paediatric population over time in a hospital.

**Method:**

A point prevalence survey of antibiotic use was performed yearly for 4 years to monitor trends in antibiotic prescribing. Data from all patients admitted before 8 a.m. on the day of the PPS were included. A web-based application designed by the University of Antwerp was used for data entry, validation, and analysis (http://www.global-pps.com).

**Results:**

A total of 260 children, including 90 (34.6%) neonates and 170 (65.4%) older children, were admitted during the four surveys. Overall, 179 (68.8%) patients received at least one antibiotic. In neonates, the prevalence of antibiotic use increased from 78.9 to 89.5% but decreased from 100 to 58.8% in older children. There was a reduction in the use of antibiotics for prophylaxis from 45.7 to 24.6%. The most frequently prescribed antibiotic groups were third generation cephalosporins and aminoglycosides. The most common indications for antibiotic prescription were sepsis in neonates and central nervous system infection in older children. The documentation of reason in notes increased from 33 to 100%, while the stop-review date also increased from 19.4 to 70%.

**Conclusion:**

The indicators for appropriate antibiotic prescription improved over time with the introduction of antibiotic stewardship program in the department.

## Introduction

The inappropriate use of antibiotics has been a source of concern globally, with increased use of last-resort antibiotics recorded globally between 2005 and 2015 [1–5]. The overuse and inappropriate prescription of antibiotics have led to antibiotic resistance in almost all groups of antibiotics [4–6]. This is worrisome, as the rate of development of antibiotic resistance is more rapid than the rate of discovery of new antibiotics to treat resistant infections [7]. The burden of antimicrobial resistance (AMR) is now a global issue and emerging threat; leading to an overwhelming increase in economic costs for patients, hospitals, and countries as well as increased mortality, especially in low- and middle-income countries [8, 9]. Antimicrobial resistance may not be eliminated; however, it can be reduced [10].

Resistance to multiple antibiotics, including carbapenems, by various organisms, has been documented in children, and these drug-resistant strains are the cause of morbidity, prolonged hospital stays, and mortality [11–19]. Studies have demonstrated patients, caregivers and prescribers are responsible for the misuse of antibiotics in children [6, 20–25]. It is therefore imperative to monitor and control antibiotic prescribing to ensure judicious use, prevent resistance, and reduce the cost of healthcare. Strategies to achieve this goal include the development of national antibiotic policies, the formation of antimicrobial stewardship (AMS) committees at hospitals and departmental levels, and adherence to national antibiotic guidelines.

Point prevalence surveys (PPS) have been used to monitor antibiotic use; in addition, they help to evaluate, monitor, and improve the AMS program [26, 27]. Globally, PPS conducted in 41 countries showed high rates of antibiotic prophylaxis, with two-thirds being used for medical prophylaxis [28], while a 15-year PPS in a paediatric hospital in Sweden showed a fourfold increase in total antibiotic prescriptions for prophylactic purposes without an increase in the number of patients [29]. In the United States of America, one in three children is receiving suboptimal therapy, with 50% not being captured in stewardship programs [30].

Point prevalence surveys of antibiotic use in Nigeria have shown a high rate of antibiotic prescribing (> 80%) in children, with more than 90% of antibiotics given intravenously [31–34]. These PPS were either performed in all age groups or over a short period. However, this study focuses specifically on the paediatric population, over four different periods[35,36].

The first PPS performed in our hospital showed poor prescribing habits, as demonstrated by the low percentage of indicators tested. An antimicrobial stewardship program was started in the hospital; subsequently, three additional PPS were carried out. This study aims to describe the patterns of antibiotic use among the paediatric population in a tertiary hospital in Lagos, Nigeria, over a period of 4 years. The objectives were as follows:

1. To describe the prevalence rates and indications for antibiotics prescription for paediatric patients.
2. To describe the effect of an antibiotic stewardship program on antibiotic prescribing practices in children over a 4-year period and identify areas for improvement.

## Materials and methods

### Study design and setting

This was a serial cross-sectional study conducted over 4 years (2015, 2017, 2018, and 2019 in the Paediatrics Department of the Lagos University Teaching Hospital located in Lagos, Southwest Nigeria. The state is estimated to have a population of more than 20 million people, with more than 50% within the paediatric age group and estimated to have 5 million under-fives [37]. It is a coastal city with a major seaport, metropolitan and cosmopolitan area, consisting of residents from Nigeria and other neighbouring African countries. The hospital is a foremost tertiary centre that is a referral centre for secondary health facilities within and for neighbouring states. There are several secondary health centres in Lagos with 3 tertiary centres providing care for children; however, the study site caters to most of the referrals because it has the largest capacity for the care of children, with a bed capacity of 160, comprising two neonatal wards, three paediatric medical wards, and an emergency room. The neonatal unit has both the inborn (for the neonates delivered in the hospital) and out-born (neonates brought from outside the hospital) wards. Children from birth to 17 years of age are seen and managed. The hospital participated in the global PPS in 2015 and subsequently in 2017, 2018, and 2019, with the paediatrics department involved. There was no global PPS conducted in 2016.

As part of the AMS programme, which started in the hospital following the initial PPS, the Department of Paediatrics AMS team was formed in 2017. The team developed its antibiotic guidelines. These were circulated to all prescribers in the department for review. The final document was adopted after a presentation to the department. The process took over a year, and the guidelines were circulated in late 2018. In addition, an antibiotic policy document was developed by the hospital AMS committee and circulated to all departments. All prescribers were expected to adhere strictly to this protocol and guideline. The Department of Paediatrics also chose prospective audit, with intervention and feedback as an AMS strategy [38], for the department.

### Study population

On a predetermined day for the global PPS, all records of paediatric patients admitted before 8 am were reviewed, and relevant information was extracted. All patients admitted to the paediatric wards who were receiving antibiotic (both oral and parenteral) medications were surveyed.

Subsequent PPS was performed to monitor trends in antibiotic prescribing, and in all the surveys, data for all patients admitted before 8 a.m. on the day of the PPS were included. The survey was conducted in all the paediatric wards over 2 weeks in the last quarter of each year. On each survey day, the PPS will be completed in at least one ward. The study population included patients aged 0–17 years and included neonates and older children.

### Data collection and analysis

The data were collected from patients’ folders using the global PPS data collection form. The clinical diagnosis was also categorized based on the global PPS. The following data were obtained from the case notes: ward name, patient identifier survey number, age, sex, antibiotic name (generic), indication for antibiotic, dose, duration of the antibiotics given, clinical diagnosis (using diagnostic code), and microbiological data. Community-acquired infection (CAI), was defined as an infection acquired in the community if the patient had not recently been in a healthcare facility or been in contact with someone, who had been recently in a healthcare facility. Hospital care-associated infection, (HAI) was defined as, a fever that started 48 h after hospital admission (1–6). Antibiotic quality indicators such as compliance with antibiotic guidelines, documentation of reasons for antibiotic prescription, route of administration, and stop and review date of prescription were also collected by trained data personnel using a standard PPS form [39]. The initial classification was based on the Anatomic therapeutic chemical classification system (ATC) based on a generic name and anatomic site of function [40]. A web-based application designed by the University of Antwerp was used for data entry, validation, and analysis http://www.global-pps.com.

### Data analysis

The data were entered into Microsoft Excel (2017) and analysed using SPSS version 20.0 The data were presented as percentages or proportions. The antibiotics prescribed were further analysed and classified as ‘Access’, ‘Watch’, or ‘Reserve’ using the WHO AWaRe classification list [41, 42]. Compliance with the guidelines and hospital antibiotic policy was also assessed. The chi-square test for trend was used to assess differences in the prevalence of antibiotic prescription and differences in quality indicators across the four PPS. The level of statistical significance was set at *p* < 0.05.

## Results

### Hospital and patient characteristics

Two hundred and sixty children, including 90 (34.6%) neonates and 170 (65.4%) older children, were admitted during the four surveys in 2015, 2017, 2018, and 2019. The number of inpatients admitted increased from 39 in 2015 to 49 in 2017, 85 in 2018, and 87 in 2019.

### Prevalence of antibiotic use

Overall, 179 (68.8%) patients received at least one antibiotic on the day of the survey. A total of 72.2% (65/90) of the patients were neonates, and 67.1% (114/170) were older. Table 1 provides the general patient characteristics and antibiotic use for the survey periods. The prevalence of antibiotic use generally decreased from 89.7% in 2015 to 65.5% in 2019 (*p* = 0.01). In neonates, the prevalence of antibiotic use decreased from 78.9% in 2015 to 36.8% in 2017 and then started to increase until 2019 (*p* = 0.001). In older children, the prevalence of antibiotic use significantly decreased from 100% in 2015 to 58.8% in 2019 (*p* = 0.004).

Most of the patients on antibiotics were prescribed more than one antibiotic, but there was no significant difference between 2015 and 2019 (Table 1). Most of the antibiotics were administered intravenously, with an average of 91.6%. There was no significant difference in the proportion of patients who received intravenous antibiotics over the 4 years. All the neonates were administered antibiotics intravenously.

**Table 1:** General characteristics of the patients, antibiotic use, and healthcare-associated infections (HAI) prevalence

Characteristic	2015(%)	2017 (%)	2018 (%)	2019 (%)	*P* value
Number of inpatients	39	49	85	87	
• Neonates	19(48.7)	19(38.8)	33(38.8)	19(21.8)	
• Children > 1month	20 (51.3)	30(61.2)	52(61.2)	68(78.2)	
Number of patients prescribed at least one antimicrobial (%)	35 (89.7)	29(59.2)	58(68.2)	57(65.5)	0.01*
• Male	23 (65.7)	17 (58.6)	36 (62.1)	32 (56.1)	
• Female	12 (34.3)	12 (41.4)	22 (27.9)	25 (43.9)	
• Neonates	15 (78.9)	7 (36.8)	26 (78.8)	17 (89.5)	0.001*
• Children > 1month	20 (100)	22(73.3)	32(61.5)	40(58.8)	0.004*
Parenteral therapy (per patient)	30 (85.7)	29 (100.0)	52 (89.7)	53 (93.0)	0.20
• -Neonates	15 (100)	7 (100)	26 (100)	17 (100)	1
• Children > 1month	15 (75)	22 (100%)	26 (81.3)	36 (90)	0.07
Multiple antibiotic use (per patient)	25(71.4)	19 (65.5)	47 (81.0)	36 (63.2)	0.17
• Neonates	15 (100)	4 (57.1)	22 (84.6)	15 (88.2)	0.06
• Children > 1month	10 (50)	15 (68.2)	25 (78.1)	21 (52.5)	0.08
Number of prescribed antibiotics	66	52	104	92	
• Neonates	35 (53.0)	12 (23.1)	53 (51)	35 (38.0)	
• Children > 1month	31 (47.0)	40 (76.9)	51 (49)	57 (62.0)	
Antibiotic prophylaxis	16 (45.7)	12 (41.4)	22(37.9)	14 (24.6)	
Patients treated for at least one HAI	5 (12.8)	9 (18.4)	15 (17.6)	10 (11.5)	0.6
• Neonates	3 (15.8)	4 (21.1)	12 (36.4)	5 (26.3)	0.38
• Children > 1month	2 (10)	5 (16.7)	3 (5.8)	5 (7.4)	0.37

*p* < 0.05, statistically significant HAI = Healthcare associated infection

### Antibiotic use among the age groups

A total of 314 antibiotics were prescribed during the four survey periods, including 135 antibiotics for neonates and 179 antibiotics for older children. The five most common antibiotics prescribed for neonates were amikacin 51 (37.8%), cefotaxime 44 (32.6%), levofloxacin 15 (11.1%), meropenem 9 (6.7%) and metronidazole 7 (5.2%), while in older children, the five most common antibiotics prescribed were ceftriaxone 47 (26.3%), cefotaxime 26 (14.5%), amikacin 26 (14.5%), metronidazole 22 (12.3%) and cefuroxime 18 (10.1%).

### Indications for antibiotic use

**Fig. 1:**
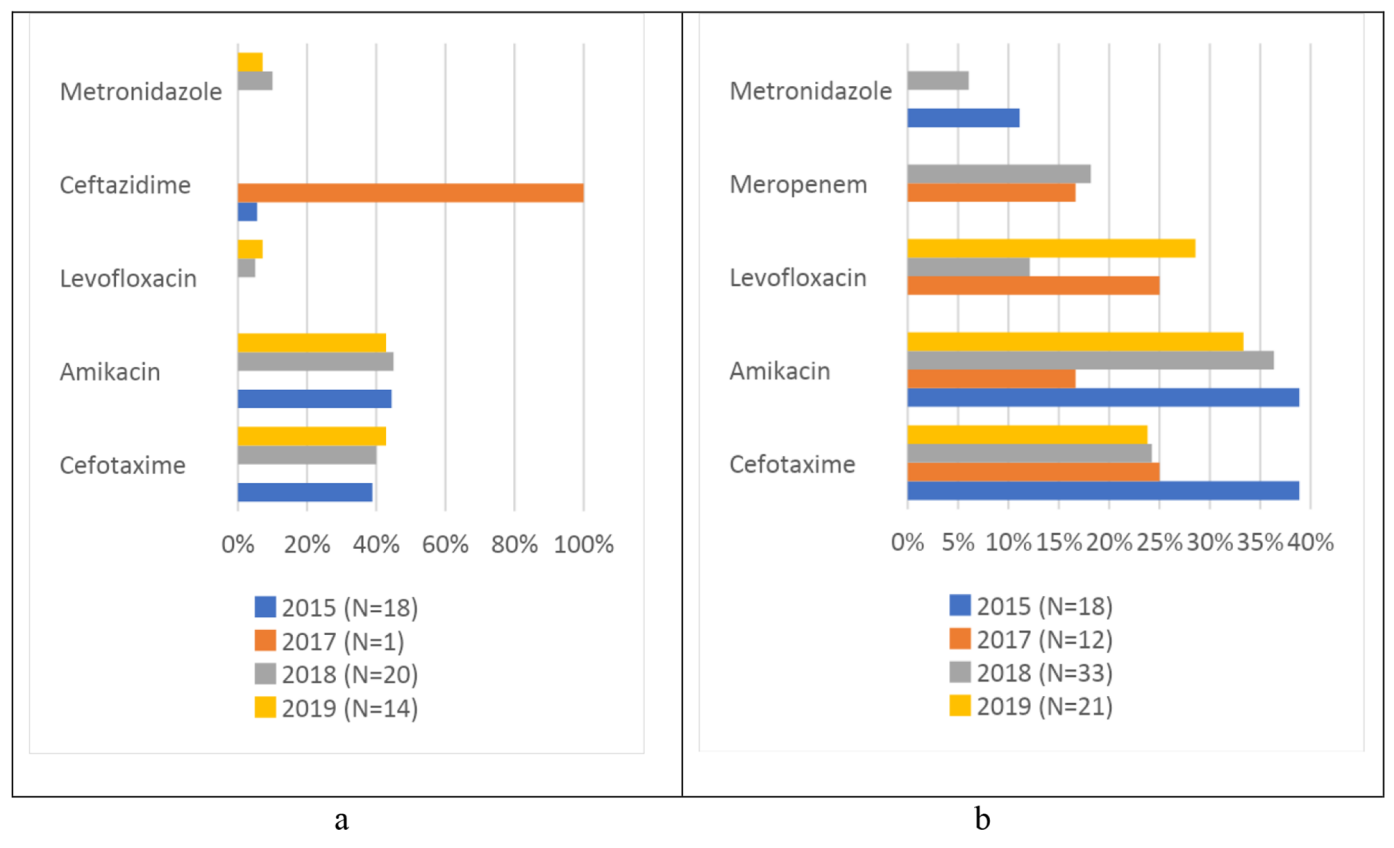
**a**: Most frequently prescribed antibiotics for medical prophylaxis in neonates. **b**: Most frequently prescribed antibiotics for therapeutic use in neonates

**Fig. 2:**
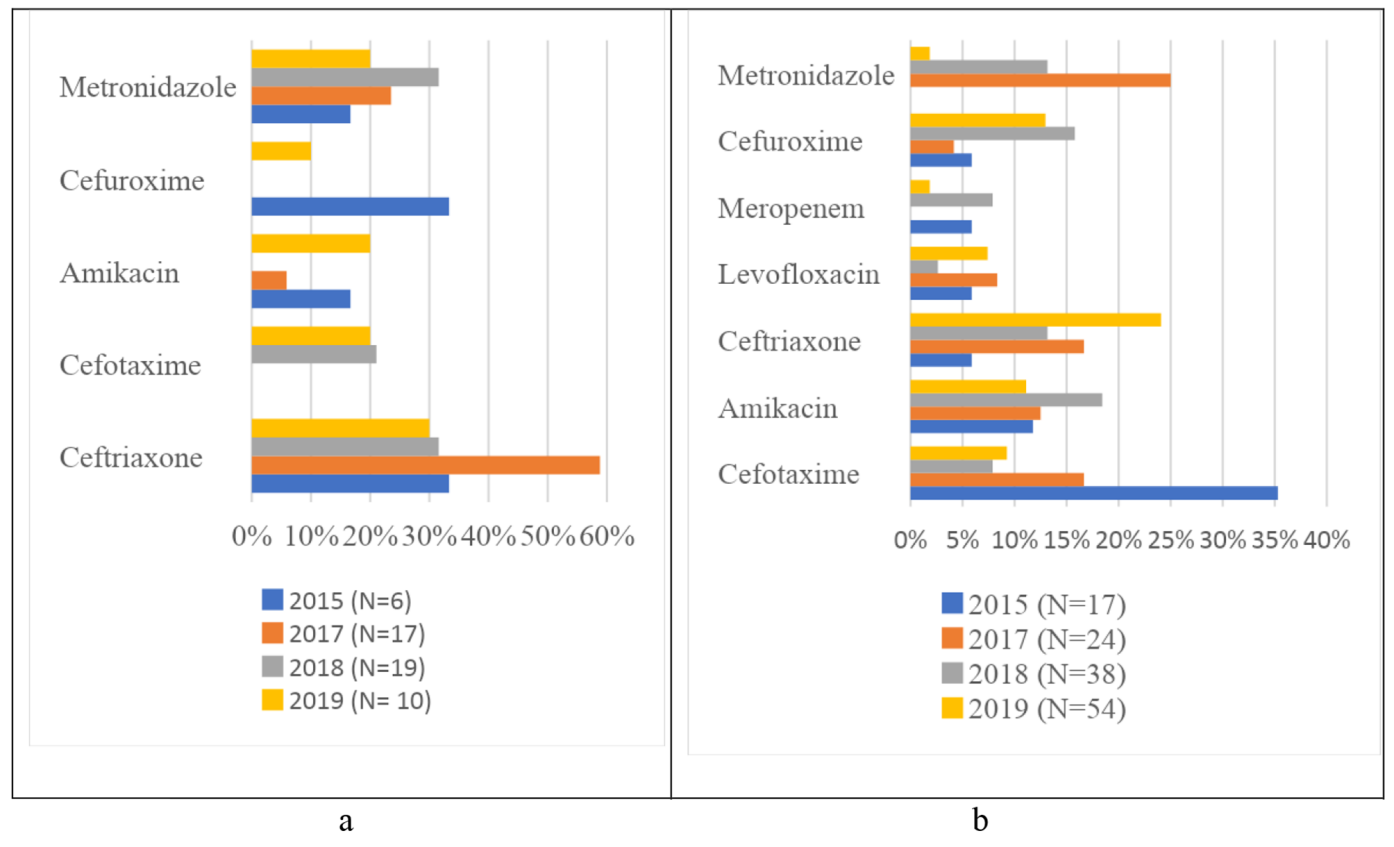
**a**: Most frequently prescribed antibiotics for surgical prophylaxis in older children. **b**: Most frequently prescribed antibiotics for therapeutic use in older children

Among the 135 antibiotics prescribed for neonates over the 4 years, 42 (31.1%) were prescribed for community-acquired infections, 41 (30.4%) for healthcare-associated infections, and 52 (38.5%) for prophylaxis (Table 2). Among the older children, 90 (50.3%) were prescribed for community-acquired infections, 26 (14.5%) for healthcare-associated infections, 59 (33%) for prophylaxis, and 4 (2.2%) for unknown reasons (Table 2). In neonates, the top three antibiotics prescribed for CAI and HAI combined were amikacin (33.7%), cefotaxime (27.7%), and levofloxacin (15.7%), while for medical prophylaxis, the top three were amikacin (44.2%), cefotaxime (40.4%) and metronidazole (5.8%) (Fig. 1a and b). In older children, the top three antibiotics prescribed for CAI and HAI combined were ceftriaxone (19.8%), cefotaxime (15.5%), and amikacin (15.4%), while for surgical prophylaxis, the top three were ceftriaxone (41.2%), metronidazole (23.5%) and amikacin (11.8%) (Fig. 2a and b).

**Table 2:** Indications for prescribing antibiotics

Characteristics	2015 (%)	2017 (%)	2018 (%)	2019 (%)	Total (%)
**Neonates**	**35**	**12**	**53**	**35**	**135**
Community-acquired infection	11 (31.4)	5 (41.7)	13 (24.5)	13 (37.1)	42 (31.1)
Healthcare associated infection	7 (20)	6 (50)	20 (37.7)	8 (22.8)	41 (30.4)
Medical prophylaxis	17 (48.6)	1 (8.3)	20 (37.7)	14 (40)	52 (38.5)
**Older child**	**31**	**40**	**51**	**57**	**179**
Community-acquired infection	12 (38.7)	12 (30)	28 (54.9)	38 (66.7)	90 (50.3)
Healthcare associated infection	3 (9.7%)	11 (27.5%)	4 (7.8%)	8 (14%)	26 (14.5)
Medical prophylaxis	7 (22.6)	0	0	1 (1.8)	8 (4.5)
Surgical prophylaxis	5 (16.1)	17 (42.5)	19 (37.2%)	10 (17.5%)	51 (28.5)
Unknown	4 (12.9)	0	0	0	4 (2.2)

Based on the diagnosis of infections, in neonates, antibiotics were mainly prescribed for neonatal sepsis and medical prophylaxis, while in older children, antibiotics were mainly prescribed for sepsis, central nervous system infections, bone and joint infections, and urinary tract infections (Table 3).

**Table 3:** Diagnosis for antibiotics prescribed in neonates and older children

Diagnoses	2015	2017	2018	2019
**Neonates**	**(** ***N*** ** = 15)**	**(** ***N*** ** = 7)**	**(** ***N*** ** = 28)**	**(** ***N*** ** = 19)**
Sepsis	5 (33.3%)	5 (71.4%)	12 (42.8%)	12 (63.2%)
Prophylaxis in neonate	7 (46.7%)	1 (14.3%)	9 (32.1%)	7 (36.8%)
Others	3 (20)	1 (14.3%)	7 (25.1%)	0
**Older children**	**(** ***N*** ** = 20)**	**(** ***N*** ** = 23)**	**(** ***N*** ** = 37)**	**(** ***N*** ** = 45)**
Sepsis	8 (40%)	13 (56.5%)	1 (2.7%)	11 (24.4%)
CNS	4 (20%)	1 (4.3%)	12 (32.4%)	11 (24.4%)
SSTBJ	3 (15%)	4 (17.4%)	4 (10.8%)	10 (22.2%)
UTI	4 (20%)	0	1 (2.7%)	1 (2.2%)
GI infection	1 (5%)	5 (21.7%)	12 (32.4%)	6 (13.3%)
Others	0	0	7 (18.9%)	6 (13.3%)

CNS = Infection of the central nervous system; SSTBJ = Skin & Soft Tissue, Bone Joint Infection = Cellulitis, wound including surgical site infection, deep soft tissue not involving bone, e.g., infected pressure or diabetic ulcer, abscess Septic arthritis (including prosthetic joint), osteomyelitis; Sepsis = sepsis, sepsis syndrome or septic shock with no clear anatomic site; Prophylaxis in neonates = Drug is used as Medical Prophylaxis for new-born risk factors, e.g., VLBW (Very Low Birth Weight) and IUGR (Intrauterine Growth Restriction) and maternal risk factors; GI inf = GI infections (*salmonellosis, Campylobacter, parasitic, C. difficile*, etc.), intraabdominal sepsis including hepatobiliary, intra-abdominal abscess, etc.; UTI = urinary tract infection; lower urinary tract infection, upper urinary tract infection including catheter-related urinary tract infection, pyelonephritis

### Use of quinolones

**Fig. 3:**
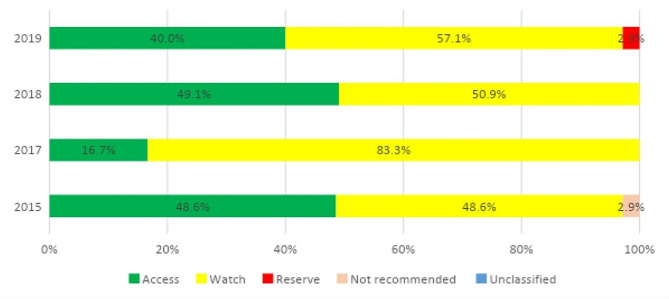
Proportion of total antibiotic (ATC J01) use in neonates according to the AWaRe classification

**Fig. 4:**
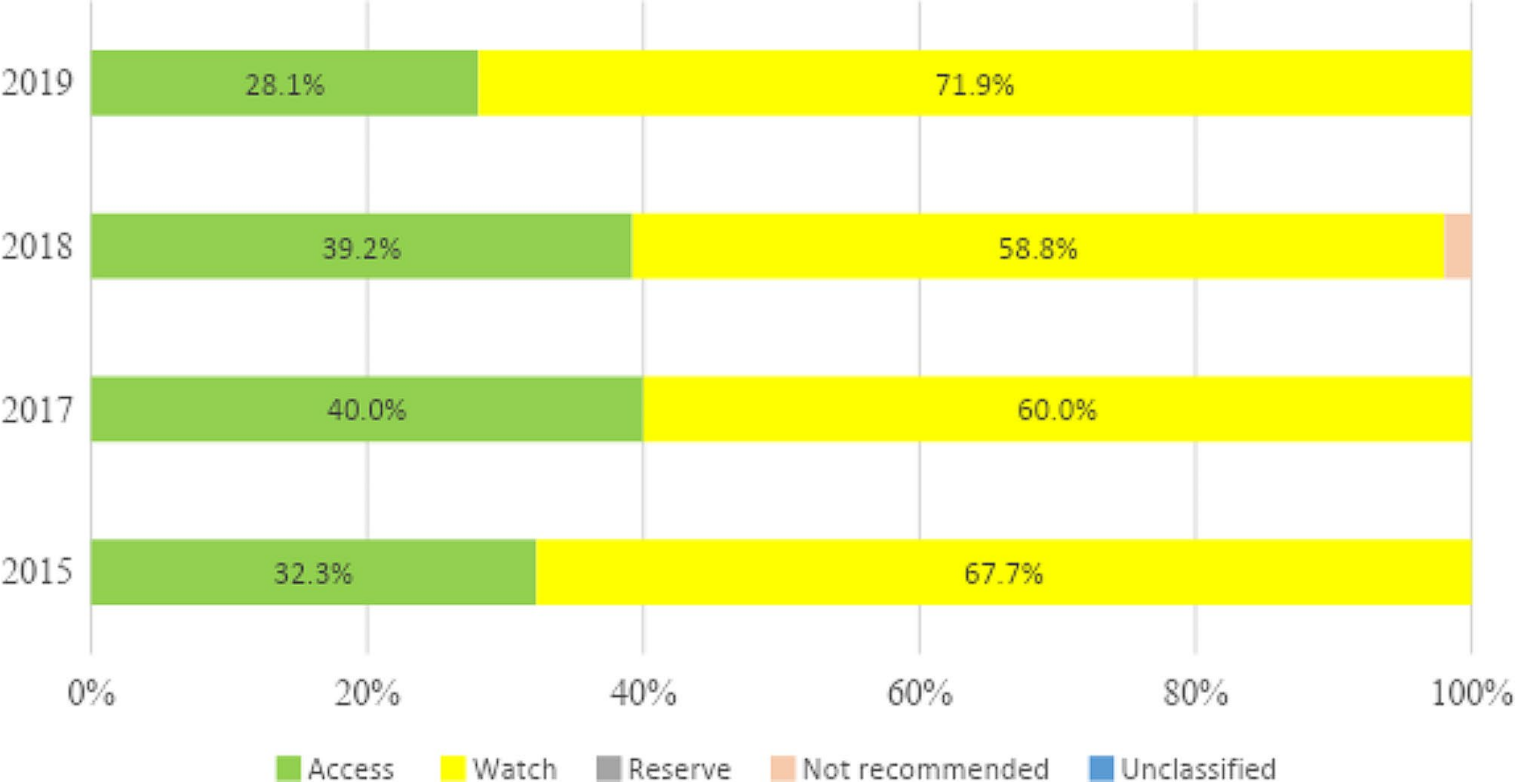
The proportion of total antibiotic (ATC J01) use in older children according to the AWaRe classification

A total of 24 prescriptions of quinolones were made over the 4 years, 15 (62.5%) in neonates. The most common indication for quinolone prescription in neonates was healthcare-associated infection (66.7%), but for older children, it was community-acquired infection (77.8%). The most common diagnosis for the prescription was sepsis in neonates. There was a high use of antibiotics in the watch group (Figs. 3 and 4).

### Quality indicators

There was a general trend in favour of improvement in the quality indicators for paediatric and neonatal wards. The prevalence of antibiotic prescriptions increased from 36.4% in 2015 to 100% in 2019 (*p* < 0.0001), and the percentage of reviews increased from 19.7% in 2015 to 92.3% in 2018 and then decreased to 67.4% in 2019 (*p* < 0.0001) (Table 4). Compliance with guidelines decreased significantly from 97.1% in 2015 to 67.2% in 2019 (*p* < 0.0001). Surgical antibiotic prophylaxis was given for more than 24 h for all patients who underwent surgery. Targeted therapy increased significantly from 3% in 2015 to 14.1% in 2019, and the increase was mainly among neonates (*p* = 0.004) (Table 4).

**Table 4:** Quality indicators for antibiotic prescribing

Characteristic	2015 (%)	2017 (%)	2018 (%)	2019 (%)	
Number of prescribed antibiotics	66	52	104	92	
Reason for antibiotic prescription in notes	24 (36.4)	21 (40.4.9)	104 (100)	92 (100%)	< 0.0001*
• Neonates	20 (57.1)	2 (16.7)	53 (100)	35(100)	< 0.0001*
• Children > 1month	4(12.9%)	19 (47.5)	51 (100)	57 (100)	< 0.0001*
Stop/review date in notes	13 (19.7)	2 (3.8)	96 (92.3)	62 (67.4)	< 0.0001*
• Neonates	11 (31.4)	0	47 (88.7)	29 (82.9)	< 0.0001*
• Children > 1month	2 (6.5)	2 (5)	49 (96.1%)	33 (57.9%)	< 0.0001*
Targeted therapy	2 (3.0)	0	14 (13.5)	13 (14.1)	0.004*
• Neonates	1 (2.9)	0	11 (20.8)	9 (25.7)	0.015*
• Children > 1month	1 (3.2)	0	3 (5.9)	4 (7)	0.38
Surgical-antibiotic prophylaxis > 24 hrs	4 (100%)	11 (100%)	10 (100%)	6 (100%)	-
• Neonates	0	0	0	0	-
• Children > 1month	4 (100%)	11 (100%)	10 (100%)	6 (100%)	-
Guideline compliance	0	0	101 (97.1)	62 (67.2)	< 0.0001
• Neonates	0	0	53 (100)	33 (94.1)	0.08
• Children > 1month	0	0	48 (94.1)	29 (50.9)	< 0.0001

*p* < 0.05, statistically significant

## Discussion

The study showed the overall prevalence of antibiotic use in children (both neonates and older children) was greater than that recommended; more so even greater than that reported in European countries [28, 43] however, it is similar to reports of other studies in Nigeria and Africa [32, 44] Therefore, if we are to gain meaningful control of antibiotic use, we must focus on children and neonates, especially in Africa. The significantly greater decrease in the prevalence of antibiotic use in older children is attributed to the implementation of the AMS program in the hospital with the development of antibiotic guidelines and compliance with its use in the Department of Paediatrics.

The increased use of antibiotics, especially in inborn neonatal wards, could be primarily due to recent advancements and improvements in facilities with the acquisition of more modern equipment as well as the increased capacity of the newborn unit to care for more preterm low weight babies, resulting in more referrals of preterm infants from other centres. It is known that a good number of preterm babies develop sepsis, especially in low and medium-income countries (LMIC) where there are poor antenatal and delivery services, with home delivery in an unhygienic environment and poor supervision during delivery. Moreso, preterm neonates tend to stay longer in the hospital and hence are more prone to HAI.

The antibiotic recommended in our guidelines for the treatment of sepsis in neonates was combination therapy with a third-generation cephalosporin and an aminoglycoside; based on a local antibiogram conducted before the start of PPS. However, it differs from the WHO recommendation for the treatment of neonatal sepsis, which involves the use of ampicillin and gentamicin, based on surveys of 56 paediatric hospitals worldwide [45, 46]. These antibiotics are also the most commonly prescribed for neonates in African hospitals [45, 47], and it reflects the resistance profile of relevant pathogens in our hospitals [48]. Continuous surveillance and antibiograms should be conducted because the current trend may not be what was previously obtained.

The decreased rate of overall use of prophylactic antibiotics is attributed to increased awareness and adherence to guidelines [49, 50] In children, suspected central nervous system infection and sepsis are very common life-threatening infections that requires antibiotics prescriptions [32, 45, 47]. Lack of access to affordable and rapid diagnosis may be the driving force as patients need to pay out of pocket for blood cultures.

High rates of medical prophylaxis, occurred in neonates, such as in other parts of Africa [32, 44]. One possible explanation for this prophylaxis is that it was used for a diagnosis of suspected sepsis in full-term newborns with major risk factors for sepsis. The antibiotic prophylaxis in neonates was combination therapies (except in 2017), including a third-generation cephalosporin and an aminoglycoside, which contrasts with our local guideline’s recommendation of second-generation cephalosporin as well as the WHO recommendation for penicillin and an aminoglycoside [45, 51, 52]. Absence of documented indications for use may be the reason for assigning them as prophylaxes, since a follow-up was not part of the PPS, the final reason cannot be ascertained. There needs to be more engagement with prescribers for newborns specifically to adhere to documented guidelines. Research on the needs and outcomes of antibiotic prophylaxis in children, especially neonates, is needed to improve rational antibiotic prescribing.

The gold standard for identifying sepsis is blood culture, which may not be affordable and may take more than 24 h to obtain a result in the LMIC. Due to ethical concerns, in centres where patients pay out of pocket for a service, and where there is a very high probability of severe and life-threatening complications from infections, an antibiotic delay is usually avoided. This calls for more affordable modern, rapid, and accurate diagnostic tests to quickly distinguish babies who have sepsis from those who do not. Thus, conserve and avoid the unnecessary use of antibiotics.

In older children, even though the rate of antibiotic use for prophylaxis decreased significantly; the use of third-generation cephalosporins is still a challenge due to its variance to both local and international guidelines. Ceftriaxone and other third-generation cephalosporins, often in combination with metronidazole for more than 24 h, are commonly observed in Nigeria and East Africa [34, 53, 54]. Prolonged surgical prophylaxis with broad-spectrum antibiotics can increase the risk of antibiotic resistance [53]. Appropriate surgical antibiotic prophylaxis is potentially a low-hanging fruit for improving the quality of antimicrobial prescribing in children. The AMS team presently includes surgeons. There is currently a departmental drive, where the team engages the surgeons regularly to inform and educate them on appropriate surgical prophylaxis and liaise with the pharmacy to make it available. The pharmacy department is at the forefront of our stewardship programs, recommended surgical prophylaxis would be made available and dispensed in surgical packs. More enforcement is needed for strict adherence to pre-authorisation. The AMS member in surgery also ensures compliance by constant reminders.

Parenteral antimicrobial therapy in all periods was similar to rates previously documented in studies in Nigeria, other African countries, East and South Asia, and North America from the Global –PPS network [34, 55–57]. An AMS intervention using an intravenous (IV) to oral antibiotic switch is a low-hanging fruit to consider in our hospitals to reduce the high rate of parenteral therapy, especially in older children. The IV-to-oral switch for older children as a supplementary strategy can help to reduce the length of hospital stay, the cost of healthcare, staff workload, and the risk of catheter-associated infections [26–28]. We currently advocate for early discharge and ambulatory care for children to reduce HAI.

### AWaRe antibiotics

The high use of “Watch” antibiotics may reflect high resistance rates to antibiotics, as shown by our antibiotic guidelines. Setting a target of 60% for access group antibiotics as recommended by the WHO would require strengthening our AMS program to reverse the trend of irrational antibiotic use [41, 58].

Notably, quinolones were frequently used in the neonates. Quinolones are contraindicated in children except for those with shigellosis or salmonellosis, immunocompromised children with multidrug-resistant organisms, and those for whom there are no other safe or effective antibiotics [59–62]. However, none of these diagnoses were made in the children. Quinolone has been used extensively in Asia and Europe even beyond its licensed indications for use [60, 62–66], especially in Belgium, where it was found to be prescribed off-label and did not follow bacteriological findings [60]. Although contraindicated, reasons for use in children include local antibiogram results and poor response to other conventional antibiotics, as studies have shown less toxicity in humans [62, 67]. This is even more worrisome, especially as organisms will become resistant to quinolones. Intervention programs targeted at minimizing the use of quinolone as they are medications in the W.H.O reserve group are urgently needed. There is a current target of reducing this in children as AMS time is allocated every week in the paediatrics department to educate and inform prescribers on AMS and the importance of using the antibiotic guidelines.

The HAI rates in neonates doubled, which was attributed to more admissions of preterm neonates with prolonged hospital stays and prolonged use of invasive procedures such as the insertion of central catheters and ventilatory support. These HAI rates are similar to the rates found in northern Nigeria (14.3%) [68], implying that a sustained infection prevention and control (IPC) program is desirable in Nigeria to attain lower rates, as recorded in Ghana (8.2%) [69] and the USA (2.98–3.13%) [70]. We suggested and activated more robust and sustained IPC within the hospital AMS program, emphasizing HAIs.

## Conclusion

Antimicrobial prescription rates were generally reduced with the hospital AMS program. However, there is still extensive use of antibiotics in our children, with the use of multiple antibiotics and reserve antibiotics, with the major diagnosis being sepsis. Cephalosporin was the antibiotic most often prescribed. There should be urgent and intensive interventions to reduce this risk.

### Recommendation

AMS programs should be enforced to curtail and reduce the unnecessary use of antibiotics. There should be continuous education and training of healthcare professionals on the use of the guidelines and diagnostic tests before the commencement of antibiotics to avoid the irrational use of antibiotics. The AMS team is currently involved in continuing professional education activities by training and giving talks on stewardship to all cadres of health workers. At each weekly departmental meetings, a five-minute talk on the rational use of antibiotics and at every other opportunity is given. Posters on AMS are also pasted at strategic places within the hospital to remind prescribers. Other AMS strategies, such as preauthorization and prospective audit feedback and intervention, have been included, as these strategies could have synergistic effects on reducing antibiotic use. The AMS team has also embarked on AMS in the outpatient and is currently mentoring peripheral hospitals.

### Limitations of the study

A point prevalence survey is a one-point analysis and may not provide a true picture of the actual situation. Other details of the use of antibiotics cannot be accessed by this method; however, multiple PPS may also be a good indication of the prevailing situation. A study on the prospective audit intervention and feedback would complement PPS to give more information on the effectiveness of AMS activities within the hospital. The study was based on in-patients only, an outpatient prescription survey would expand the scope of knowledge on antibiotic use.

## Data Availability

The datasets used and/or analysed during the current study will be available from the corresponding author upon reasonable request.

## Abbreviations

A.T.C: Anatomic therapeutic chemical classification system
AMR: Antimicrobial resistance
AMS: Antimicrobial stewardship
AWaRe: ‘Access’, ‘Watch’, and ‘Reserve’
CAI: Community-acquired infection
CNS: Central nervous system
GI inf: GI infections
HAI: Healthcare associated infection
IPC: Infection prevention and control program
IUGR: Intrauterine Growth Restriction
MDRO: Multidrug-resistant Organisms
PPS: Point Prevalence Survey
SAP: Surgical antibiotic prophylaxis
SSTBJ: Skin & Soft Tissue, Bone Joint Infection
UTI: Urinary Tract Infection
VLBW: Very Weight low birth weight
W.H.O: World Health Organization
